# Construction of fatty acid derivatives from rubber seed oil as α-glucosidase inhibitors based on rubber seed oil

**DOI:** 10.1186/s40643-022-00492-9

**Published:** 2022-03-21

**Authors:** Jiahao Liu, Renwei Zhang, Kaili Nie, Changsheng Liu, Li Deng, Fang Wang

**Affiliations:** 1 Beijing Bioprocess Key Laboratory and State Key Laboratory of Chemical Resource Engineering, College of Life Science and Technology, Beijing University of Chemical Technology (BUCT), Beijing, 100029 People’s Republic of China; 2 Sinovac Biotech Ltd, Beijing, China

**Keywords:** α-Glucosidase, Rubber seed oil, Hydration, Esterification

## Abstract

Natural free fatty acids show inhibitory effects on α-glucosidase and can hence have potential applications in diabetes treatment. This study indicated that the inhibitory effect of fatty acids showed a significant negative correlation with affinity energy (− 0.87) and melting point (− 0.88). Guided by this relationship, two promotion strategies of hydration and esterification were put forward to increase the inhibitory effect of fatty acids on α-glucosidase. The hydration can import an extra hydroxy group into the C=C bond of fatty acids, that will enhance the interaction with α-glucosidase, while the esterification will lower the melting point of fatty acids, and promote the inhibitory effect. Hydroxy fatty acids and fatty acid isopropyl esters possessed higher inhibitory effects than the natural fatty acids. Then, rubber seed oil was modified into novel fatty acid derivatives with higher inhibitory effect on α-glucosidase. The inhibitory IC50 of hydroxy products and isopropanol esters was 0.42 ± 0.01 μM and 0.57 ± 0.01 μM, respectively. The result reveals a feasible route to construct fatty acid derivatives from natural oil with α-glucosidase inhibitory effect.

**Graphical Abstract:**

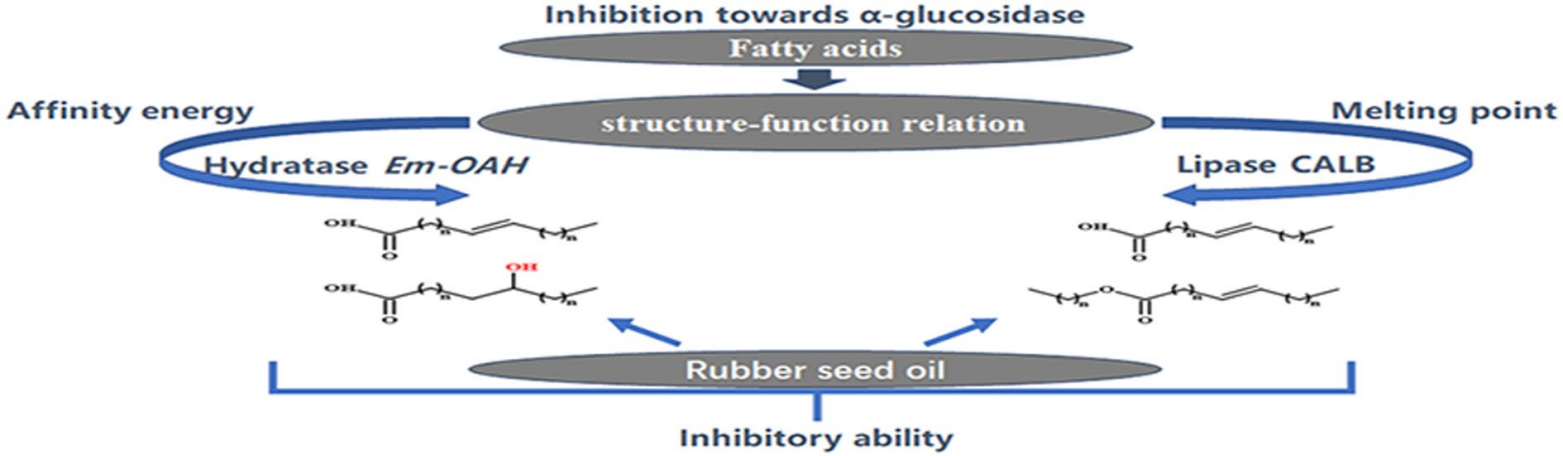

**Supplementary Information:**

The online version contains supplementary material available at 10.1186/s40643-022-00492-9.

## Introduction

Diabetes has become a big challenge for the global health system and brings high costs of medicines for patients (Chatterjee et al. 2017). It is estimated that by 2045, the population of diabetes patients will reach 693 million (Cho et al. 2018), and long-term diabetes will lead to chronic damage and dysfunction of various tissues, especially eyes, kidneys, heart, blood vessels and nerves (Deedwania 2018). α-Glucosidase inhibitors, which prevent postprandial hyperglycemia by delaying the digestion of carbohydrates in the gut, are usually employed for controlling postprandial blood glucose levels (Xu et al. 2019). Many non-sugar products from the natural sources perform well in inhibiting the activity of α-glucosidase, drawing tremendous attention for their abundant source and good biocompatibility (Mata et al. 2013), such as terpene, alkaloid, quinine, flavonoid, phenol, phenylpropanoid, as well as organic acids and ester (Kumar et al. 2011; Zy et al. 2014).

At the same time, lots of seed oils have been found as an effective α-glucosidase inhibitor (Teng and Chen 2016). Kutoh et al. reported the dietary omega-3 polyunsaturated fatty acids and their metabolic derivatives are potential drugs to prevent and treat diabetes (Kutoh et al. 2021). Chen and Kang reported that the hexane extract from oriental melon seeds was an effective inhibitor for α-glucosidase with 35.30% inhibition rate at 2 mg/mL (Chen and Kang 2013). Pi et al. found that methanol extracts of *Arctium lappa L.* showed a good inhibition towards α-glucosidase with the IC_50_ of 30 μmol/mL (Pi et al. 2010) and the inhibitory compounds were identified as methyl palmitate, methyl linoleate and methyl linolenate. They further revealed that fatty acids with double bond had stronger inhibitory ability (Miyazawa et al. 2005). Among the common fatty acids, oleic acid, linoleic acid and linolenic acid possessed higher inhibitory effects towards α-glucosidase than others (Su et al. 2013).

Free fatty acids (FFA) and their derivates seem to be attractive potential α-glucosidase inhibitors to treat diabetes. Previous studies on the inhibition ability of various natural FFAs on the α-glucosidase showed that FFAs with longer carbon chain and more C=C bonds performed a higher inhibition effect (Teng and Chen 2016). However, the inhibitory effect of free fatty acids on α-glucosidase needs to be improved to enhance its application potential in medicine at present. Hence, developing proper FFA derivates with good inhibitory effects is attractive. In this paper, the structure–activity relationship was investigated by analyzing the inhibitory performance and their properties of various FFAs (physical properties and virtual molecular affinity energy). Then two strategies, hydration and esterification, were proposed to improve the inhibition ability of FFA on α-glucosidase activity. At the same time, this paper applies these two promotion strategies to rubber seed oil, a natural oil, and constructs fatty acid derivatives as improved α-glucosidase inhibitors, providing a feasible route to construct fatty acid derivatives with α-glucosidase inhibitory effect from natural oil.

## Materials and methods

### Materials

α-Glucosidase from recombinant yeast (50 units/mg), p-nitrophenyl-α-d-glucopyranoside (α-PNPG, 99%), α-linolenic acid (C18:3, 98%), and isopropyl-β-d-thiogalactoside (IPTG) are purchased from Shanghai yuanye Bio-Technology Co. Ltd. Oleic acid (OA,C18:1, 85%) and linoleic acid (LA,C18:2,85%) are purchased from TCI Co. Ltd. DHA (C22:6, 98%) is purchased from Sigma-Aldrich Co. Ltd. Caprylic acid (C8:0), capric acid (C10:0), lauric acid (C12:0), myristic acid (C14:0), palmitic acid (C16:0), stearic acid (SA,C18:0), methanol, ethanol, propanol, butanol, amyl alcohol, hexanol, octanol, isopropanol, toluene, and other reagents are analytical reagent. Novozymes 435 (*Candida antarctic* lipase B, CALB, 10,000 U/g) is purchased from Novozymes. Rubber seed oil is purchased from Xishuangbanna Huakun Biotechnology Co. Ltd.

### IC_50_ of α-glucosidase inhibition

The measurement of α-glucosidase inhibitory (IC_50_) referred to the reported method (Chen and Kang 2013). Fatty acids and their derivates were diluted in 0.1 M, pH = 6.9 phosphate buffer with 0.5% (v/v) DMSO as cosolvent, to prepare 400 mM to 0.25 mM sample solutions. 50 μL sample solution and 100 μL 0.5 U α-glucosidase (prepared in the same phosphate buffer) were added into 1.5-mL plastic tubes, incubated at 37  °C, 10 min. Then, 50 μL 5 mM α-PNPG (prepared in the same buffer) was added into the plastic tube, incubated for another 10 min at 37  °C. At the end, 1 M Na_2_CO_3_ was added to terminate the reaction. Then, 200 μL solution was taken out and added to the 96-well microplate, measured at 405 nm:

$$\% \text{inhibition} = \left({1} - \frac{ \text{Absorbance of sample} - {\text{absorbance of control}} } {{ \text{Absorbance of control} }} \right) \times 100.$$

IC_50_ was the sample concentration that inhibits the activity of 50% α-glucosidase. The Inhibitory score measures the inhibitory intensity of each sample on α-glucosidase, calculated by 1/IC_50_. Data were subjected to the one-way ANOVA analysis by SPSS 23.0, shown as mean ± SD (*n* = 3).

### Molecular docking and structure–function analysis

#### Molecular docking

The structure of α-glucosidase was obtained by the homologous modeling based on 3aj7 (Thao et al. 2021). Each compound was docking into the active site of α-glucosidase (Glu273, Asp346) by Auto Dock Vina 27 (Trott and Olson 2009). The conformation of each compound was generated to obtain the best affinity energy, in which the higher absolute value of affinity energy, the stronger protein–ligand interaction.

#### Correlation analysis and regression model

The bivariate Pearson correlation analysis was performed by SPSS 23.0; all the comparisons are made on statistical analysis of variance (ANOVA). The linear regression model applied the data analysis function of EXCEL.

### Hydration of unsaturated fatty acids

*Em-*OAH hydratase can perform asymmetric addition of water to the C=C bond at Δ9 position of oleic acid (C18:1, OA), linoleic acid (C18:2) and linolenic acid (C18:3,LA), yielding 10-hydroxystearic acid 10-HSA),10-hydroxy-cis-12-octadecenoic acid (10-HOA) and 10-hydroxy-cis-12,15-octadecenoic acid, respectively (Yan et al. 2020), and the situation of adding water to the acid given in the chemical formula is described in Additional file 1: Fig. S7. *Em-*OAH hydratase was expressed in *E. coli* BL21 and obtained after freeze-drying the cell sediment. The enzymatic synthesis of hydroxyl fatty acids was performed in 200 mL of 50 mM citrate/phosphate buffer (pH 6.5) containing 2 g FFA, 2 g freeze-dried cell, and 100 μL Tween 80 at 35 °C in a 500-mL conical flask for 20 h (Nie et al. 2020). After the reaction, 1 mL of ethyl acetate was added to the reaction system to extract hydrated products. After centrifugation at 5000 rpm, the ethyl acetate solution was collected and samples were analyzed by GC.

### Esterification of oleic acid with various alcohols

2 g (7.09 mmol) oleic acid and equimolar quantities of alcohols were added in 50-mL triangular flask. 1 g Novozyme 435 (CALB), 10 mL toluene, and 0.25 g 4A molecular sieve were added in the flask, incubated at 45 °C, 200 rpm for 24 h. CALB and molecular sieve were separated by filtration. Toluene was removed by rotary evaporation to obtain products, and samples were analyzed by GC.

### The hydrolysis of rubber seed oil and hydration of hydrolyzed fatty acids

10 g rubber seed oil, 2.3 g KOH, 4.4 mL deionized water and 26.4 mL 95% ethanol were added into a three-neck flask. The reaction was performed at 60 °C for 1 h with nitrogen reflux. At the end of the reaction, 25 mL distilled water was added, and then 30 mL n-hexane was used to extract unsaponifiable matter. The hydration layer was diluted to pH = 1 with 3 M HCl, and then free fatty acids were extracted with n-hexane. The extract was dried with anhydrous sodium sulfate and then filtered off. Then, n-hexane was removed by rotary evaporation. After methyl esterification, the sample was analyzed by gas chromatography, which was used to analyze the fatty acid composition of rubber seed oil. The measurement of acid value of rubber seed oil referred to the reported method (Khan et al. 2018). The hydration method of hydrolyzed fatty acids from rubber seed oil was the same as the previous method in 2.3.

### Esterification of rubber seed oil with isopropyl alcohol

The response surface method V8.0.5 with Design Expert was applied to optimize key factor of the esterification reaction between rubber seed oil and the isopropyl alcohol, including temperature (X1), enzyme quantity (X2) and substrate ratio (X3). The range and the levels of the test variables are given in Table 1. The products were collected and analyzed by GC–MS.

**Table 1:** Factors and levels of Box–Behnken experiments for optimization of esterification reaction conditions

Factor	− 1	0	1
Temperature (°C)	40	50	60
Enzyme quantity (%)	3	4.5	6
Substrate ratio	3	6.5	10

### Analytical procedure

#### GC analysis for hydrolysis products

The products of the hydration reaction were analyzed by GC (Shimadzu Corporation of Japan) equipped with a DB-Wax column (30 m × 0.25 mm × 0.25 μm), using nitrogen as carrier gas. The temperature of the injector was set at 240 °C. The initial temperature of the column was 60 °C, maintained for 1 min. Then the column temperature was increased to 200 °C at a rate of 25 °C/min, and maintained for 1 min. Finally, the column temperature was increased to 230 °C at a rate of 3 °C/min and maintained for 12 min. The contents of products were calculated by area normalization.

#### GC analysis for hydration products

The products of the hydration reaction were analyzed by GC (Shimadzu Corporation of Japan) equipped with a DB-1 ht column (30 m × 0.53 mm × 0.15 μm), using nitrogen as carrier gas. The temperature of the injector was set at 380 °C. The initial temperature of the column was 200 °C, then the column temperature was increased to 350 °C at a rate of 35 °C/min, and maintained for 2 min. Finally, the column temperature was increased to 380 °C at a rate of 20 °C/min and maintained for 13 min, the contents of products were calculated by area normalization.

#### GC–MS analysis for products

The products of the esterification were analyzed by GC–MS (Agilent 7890A-5975C) equipped with a HP-5 ms column (30 m × 0.25 mm × 0.1 μm, J&W Scientific Columns, Agilent Technologies), using helium as carrier gas. The temperature of the injector was set at 290 °C. The Initial temperature of the column was 50 °C, maintained for 1 min. Then the column temperature was increased to 290 °C at a rate of 6 °C/min, and maintained for 10 min. The MS was operated with an ion-source of 280 °C and 70 eV. Peaks were identified with a library search of NIST98. GC–MS results were quantified using the peak area normalization method. All measurements were conducted in triplicate. The contents of products were calculated with the method of area normalization.

## Results and discussion

### Structure function analysis of FFA on the inhibition towards α-glucosidase

The inhibitory ability of various natural FFAs on the α-glucosidase has been researched (Table S2). In order to obtain a quantitative result between the dependent variables and independent variables, two discrete variables (carbon length and C=C bond number) of fatty acids were developed into three continuous variables (affinity energy, melting point, and calculated log P (Tetko and Tanchuk 2002), shown in Table 2. At the same time, the inhibitory score (1/IC_50_) was defined as the dependent variable. Based on these continuous independent variables and one dependent variable, the quantitative research can be conducted by SPSS. The SPSS correlation analysis showed that the affinity energy had a significant negative correlation with inhibitory score at a correlation coefficient of − 0.87 (*p* < 0.01). Fatty acids with a higher absolute value of affinity energy illustrated a stronger ligand–protein interaction with α-glucosidase, bringing a stronger inhibitory effect. Rahim et al. had the similar findings that thiazole derivatives with a higher absolute value of affinity energy performed a better inhibitory ability towards α-glucosidase (Rahim et al. 2015). Besides, the melting point showed a significant negative correlation with the inhibitory score at a correlation coefficient of − 0.88 (*p* < 0.01), revealing that lowering the melting point can enhance the inhibitory ability. However, the correlation coefficient of log P (0.35, *p* = 0.30) cannot illustrate a significant association. Based on the significant correlation among the affinity, melting point and inhibition score, a linear regression model was built (Additional file 1: Table S1):

**Table 2:** The IC_50_, inhibitory score, affinity energy and melting point of various FFA

Fatty acids	IC_50_ (μM)	Inhibitory score	Affinity energy (kJ/mol)	Melting point ( °C)	log P
C8:0	98.04 ± 4.07	3.83	− 23.00	16.50	2.92
C10:0	113.64 ± 2.37	3.70	− 23.90	31.60	3.93
C12:0	135.14 ± 6.43	3.45	− 23.90	44.80	5.13
C14:0	56.18 ± 2.81	3.43	− 24.70	53.50	6.1
C16:0	13.97 ± 0.01	3.33	− 25.10	62.50	7.23
C18:0	11.34 ± 0.14	3.12	− 24.70	70.00	8.02
*cis*-C18:1	0.81 ± 0.04	4.51	− 26.40	13.00	7.68
C18:2	0.60 ± 0.01	4.99	− 27.20	− 5.00	7.11
C18:3	0.54 ± 0.01	5.34	− 28.50	− 11.00	6.65
C20:5	0.48 ± 0.01	6.70	− 32.20	− 47.40	6.83
*trans*-C18:1	ND	3.92	− 26.40	44.00	7.68

$${\text{Y}}_{\text{inhibitory score}} = -{0.18}{\text{x}}_{{\text{affinity energy}}}-{0.019}{\text{x}}_{\text{Melting point}}, {\text{r}}^{2}=0.76 \quad \left(**\right).$$

The coefficients of affinity energy and melting point are − 0.18 and − 0.019, respectively. The SPSS correlation analysis and linear regression model indicated: (1) enhancing the absolute value of affinity energy between FFA and α-glucosidase might improve its inhibitory effect; (2) lowering the melting point of FFA might strengthen its inhibitory effect; (3) the affinity energy with coefficient of − 0.18 tends to have a larger influence than the melting point with coefficient of − 0.019.

### The improvement of inhibitory ability on α-glucosidase by modified fatty acids

**Fig. 1:**
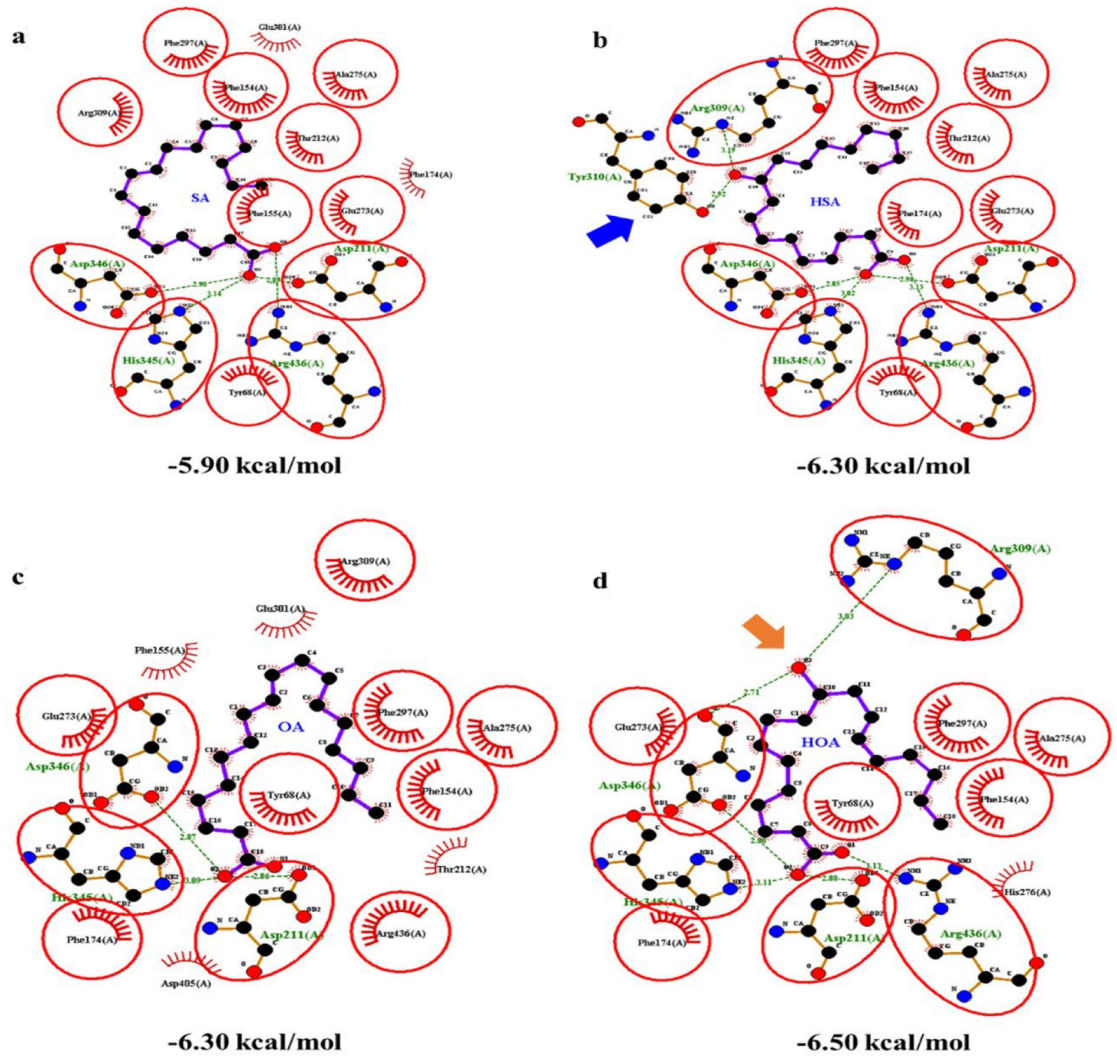
FFAs and α-glucosidase complex binding site, prepared with LigPlot+. **a** SA, **b** HSA, **c** OA, **d** HOA

Based on the linear correlation, the improvement of affinity energy absolute value will increase the inhibitory effect with a coefficient of − 0.18. Affinity energy refers to interaction between ligands and proteins. Compared with common fatty acids, the hydroxy fatty acid contains a hydroxy group, which can form an extra hydrogen bond with the cavity pocket of α-glucosidase and improve the affinity energy absolute value (Fig. 1). Compared with SA, the extra hydroxy group of 10-HSA in C10 can form two hydrogen bonds with Tyr310 and Arg309, strengthening the interaction between ligands and protein from − 24.7 kJ/mol of SA to − 29.0 kJ/mol of 10-HSA (Fig. 1a, b). Molecular docking results of OA and 10-HOA showed the similar pattern (Fig. 1c, d). The extra hydroxy group of 10-HOA in C10 can form two extra hydrogen bonds with Asp346 and Arg309, improving the affinity energy from − 26.40 kJ/mol of OA to − 27.20 kJ/mol of 10-HOA.

In the hydration reaction, to obtain a hydroxy group and strengthen the ligand–protein interaction, oleic acid and linoleic acid were hydrated by *Em-*OAH, yielding 95% 10-HSA (Additional file 1: Fig. S1) and 45% 10-HOA, respectively. 10-HOA was purified by the acetone freeze crystallization and TLC column chromatography (methanol:dichloromethane = 1:40), yielding a 94% purity (Additional file 1: Fig. S1). A significant improvement of inhibitory ability was found in 10-HSA and 10-HOA (Table 3). The extra hydroxy group improved the inhibitory score of 0.090 (SA) and 1.24 (OA) to 0.58 (10-HSA) and 2.58 (10-HOA). Paul et al. (2010) also reported that a hydroxy fatty acid, 10-hydroxy-8 (E)-octadecenoic acid, was a useful inhibitor for α-glucosidase. Our result supports that the construction of an extra hydroxy group in normal FFAs can improve their inhibitory performance.

**Table 3:** IC_50_, inhibitory score, affinity energy and melting point of hydroxy fatty acid

Fatty acids	IC_50_ (μM)	Inhibitory score	Melting point ( °C)	Affinity energy (kJ/mol)
SA	11.34 ± 0.14	3.12	70.00	− 24.70
10-HSA	1.71 ± 0.02	3.21	81.00	− 26.40
OA	0.81 ± 0.04	4.51	13.00	− 26.40
10-HOA	0.39 ± 0.01	4.79	5.50	− 27.20

#### The improvement of inhibitory ability on α-glucosidase by esterification

At the same time, the linear regression indicated that lowering the melting point might bring a higher inhibitory effect with a coefficient of − 0.019. FFA with higher melting point might have a poor interaction with α-glucosidase, since they will crystallize and precipitate at ambient temperature.

The linear regression indicated that lowering the melting point might bring a higher inhibitory effect with a coefficient of − 0.019. The esterified modification will adjust the melting point of FFA by affecting the intermolecular Van der Waals' force (Yao et al. 2008). Different alcohol modifications lead to the change of melting points. Hence, a series of oleate esters were synthesized and their inhibitory ability was measured.

After the esterification, methyl oleate, ethyl oleate, propyl oleate, butyl oleate, amyl oleate, hexyl oleate, octanol oleate, and isopropyl oleate with purity over 95% were obtained. With the increase of carbon atoms in the alcohol moiety, the melting point of oleate esters decreases sharply from 13.00 °C of OA to − 33.40 °C of isopropyl oleate and then increases to − 2.90 °C of octyl oleate, but the affinity energy absolute value of oleate esters reduced slightly (Table 4). Among all esters, isopropyl oleate possessed the lowest melting point of -33.40 °C, and relatively good affinity energy of -25.50 kJ/mol. Isopropyl oleate also showed a better inhibitory score of 1.67 and IC_50_ of 0.60 ± 0.01 μM. The improvement of inhibitory ability fits the previous finding that lower melting point contributed to a better inhibitory ability.

**Table 4:** IC_50_, inhibitory score, affinity energy and melting point of oleate esters

Fatty acids	IC_50_ (μM)	Inhibitory score	Melting point ( °C)	Affinity energy (kJ/mol)
OA	0.81 ± 0.04	1.23	4.51	− 26.40
Methyl	1.51 ± 0.05	0.66	4.76	− 24.70
Ethyl	2.44 ± 0.14	0.41	4.86	− 25.10
Propyl	1.75 ± 0.04	0.57	4.89	− 24.30
Butyl	2.34 ± 0.11	0.43	5.19	− 25.50
Isopropyl	0.60 ± 0.01	1.67	5.22	− 25.50
Pentyl	3.21 ± 0.17	0.31	5.00	− 25.10
Hexyl	4.16 ± 0.21	0.24	5.01	− 25.10
Octyl	3.25 ± 0.08	0.31	4.50	− 24.70

### The improvement of inhibitory ability on α-glucosidase modified rubber seed oil

#### The composition of fatty acids of rubber seed oil

Firstly, we illustrate the structure–function relationship of fatty acids on the inhibitory effect towards α-glucosidase. Based on the SPSS correlation analysis, which showed that the affinity energy and the melting point had significant negative correlation with inhibitory scores at a correlation coefficient of − 0.87 (*p* < 0.01) and − 0.88 (*p* < 0.01), respectively, we designed and synthesized two fatty acid derivates (hydroxy fatty acids and fatty acid isopropyl ester) with better inhibitory ability. Compared with the natural FAAs, the IC_50_ of hydrated products, 10-HSA and 10-HOA, were improved, respectively. Secondly, the esterified modification can lower the melting point of FFA and increase the inhibitory performance. Among oleate esters, isopropyl oleate possessed the best inhibitory effect with IC_50_ of 0.60 ± 0.01 μM. Rubber seed oil is a natural oil with abundant unsaturated fatty acids and the composition of fatty acids is identified in Table 5.

**Table 5:** Composition of hydrolyzed fatty acid by rubber seed oil

Fatty acids	C16:0	C18:0	C18:1	C18:2	C18:3	Acid value (KOH/g)
Proportion (100%)	9.545	6.69	28.368	37.635	16.672	2.2

#### The hydration of hydrolyzed fatty acids from rubber seed oil

The natural rubber seed oil does not have any inhibitory effect on α-glucosidase. Rubber seed oil was hydrolyzed to obtain hydrolyzed fatty acids, which then were hydrated by *Em*-OAH method. Unreacted fatty acids were removed by freeze crystallization. Hydroxyl products contain 90% hydroxyl fatty acid mixture, including 10-HSA, 10-HOA and 10-HLA (Additional file 1: Fig. S3). Compared with the natural rubber seed oil, the inhibitory ability of hydroxy fatty acids on α-glucosidase was significantly improved to 0.42 ± 0.01 μM. The results showed that the rubber seed oil which had no inhibitory effect on α-glucosidase could be modified by hydrolytic reaction and hydration reaction, and thus it had the ability to inhibit α-glucosidase.

#### Esterification of rubber seed oil with isopropanol

**Fig. 2:**
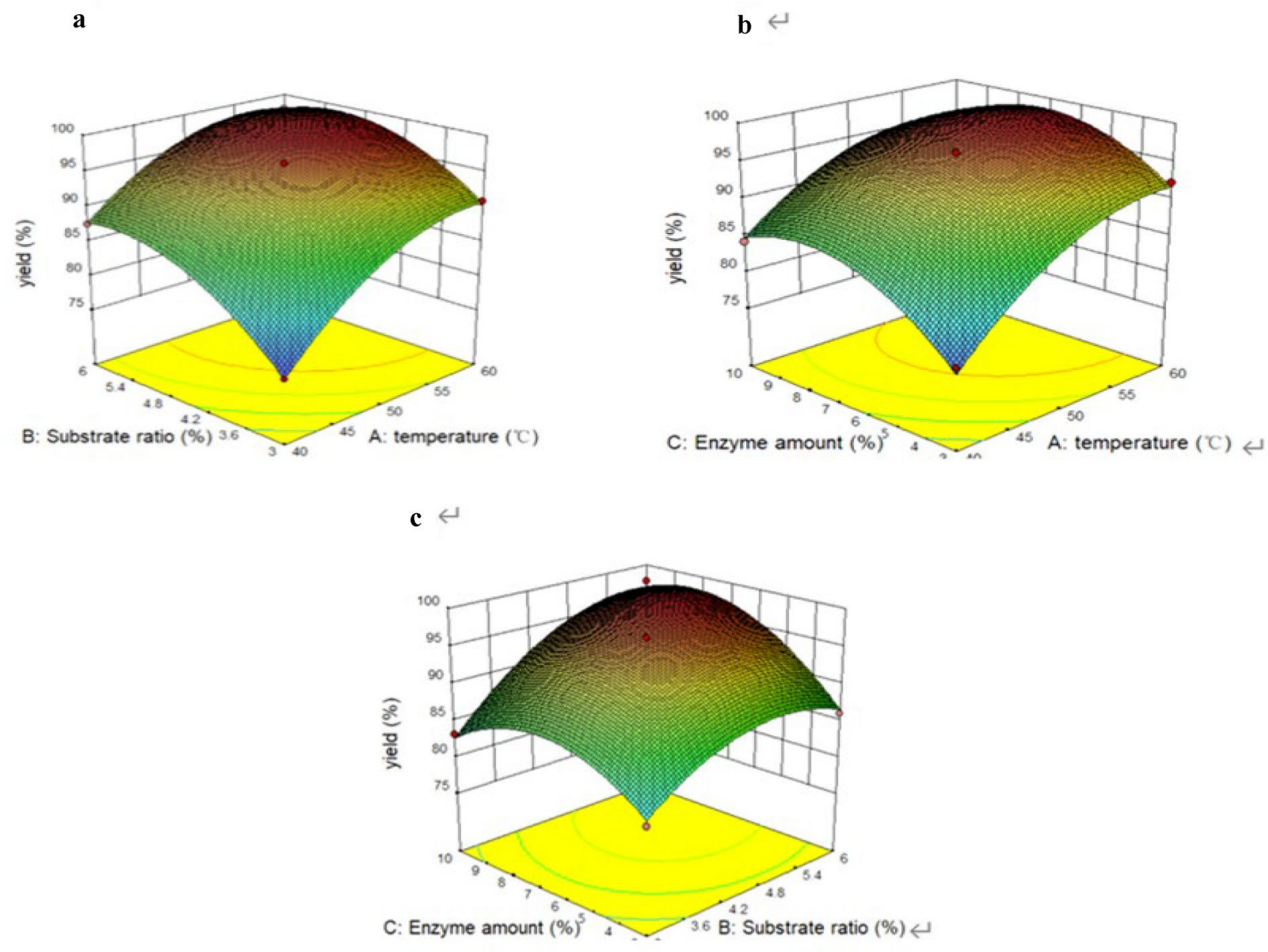
Three-dimensional response surface. **a** Effect of substrate ratio and temperature on yield; **b** effect of enzyme amount and temperature on yield; **c** effect of enzyme amount and temperature on yield

Isopropyl ester was obtained by one-step enzymatic transesterification between rubber seed oil with isopropanol. The response surface method was selected to optimize the key factors in transesterification (Additional file 1: Table S3). Through statistical analysis of variance (ANOVA), the results were analyzed and reported in Additional file 1: Table S4. The *F*-value = 109.46 implies a significant model and *R*^2^ = 0.9984 indicates a well-matched model. Besides, X1, X2, X3, X1^2^, X2^2^ and X3^2^ are significant model terms with *p*-values < 0.05, indicating that they are significant model terms. The ratio of substrates seems to have the least influence on the process, and the reaction temperature was the most influential parameter, which is expressed by the sum of squares. Three-dimensional response surface curves of different standards are given in Fig. 2, which are drawn for the yield of isopropyl fatty acid obtained in response surface design, so as to study interaction between three selected variables and determine the optimal content of each variable. The quadratic effect of temperature, enzyme quantity and substrate ratio affected the fatty acid isopropyl ester production (*p* < 0.0001), significantly. Based on the response surface, we developed a quadratic non-linear polynomial equation:

$${\text{Yield}}= 95.15+6.31 {\text{X}} 1+4.88 {\text{X}} 2+2.90 {\text{X}} 3-1.21 {\text{X}}1{\text{X}}2-1.23{\text{X}}{1}{\text{X}}3+2.27 {\text{X}}{2}{\text{X}}{3}-3.35{\text{X}}{1}^{2} -3.61{\text{X}{2}}^{2} -4.63{\text{X}{3}}^{2} \, {\text{R}^{2}} = 0.99.$$

The optimum technological parameters were determined as temperature 52 °C, substrate ratio 5.84, enzyme quantity 7.00%, based on the analysis of Design Expert, the predicted yield and the actual yield were 98.88% and 98.00%, respectively (Additional file 1: Fig. S4). The products were collected and analyzed by GC–MS, the composition of products is shown in Table 6. Compared with the natural oil, the inhibitory effect of isopropyl esters was improved significantly with IC_50_ of 0.57 ± 0.01 μM.

**Table 6:** Composition ratio of esterification reaction products

Reaction products	Isopropyl palmitate	Isopropyl stearate	Isopropyl oleate	Isopropyl linoleate	Isopropyl linolenic acid
Proportion (100%)	11.74	34.1	42.5	1.76	9.9

## Conclusion

The result reveals that hydration and isopropyl esterification are useful strategies to improve the inhibitory of FFAs towards α-glucosidase. Then, the hydration and esterification strategy were applied for rubber seed oil to construct fatty acids derivatives with better α-glucosidase inhibition. Both the hydration and esterification strategy can convert the rubber seed oil with almost zero α-glucosidase inhibition into two effectively fatty acids derivatives, with IC50 of 0.42 ± 0.01 μM for hydroxy product and 0.57 ± 0.01 μM for isopropanol esters. This paper reveals a feasible route to construct fatty acid derivatives from α-glucosidase inhibitory effect from natural oil.

### Supplementary Information

**Additional file 1.** Supporting information.

## Data Availability

All data generated or analyzed during this study are included in this published article [and its Additional file].

## References

[CR1] Chatterjee S, Chatterjee S, Khunti K, Khunti K, Davies MJ, Davies MJ (2017). Type 2 diabetes. Lancent.

[CR2] Chen L, Kang YH (2013). In vitro inhibitory effect of oriental melon (*Cucumis melo* L. var. *makuwa* Makino) seed on key enzyme linked to type 2 diabetes. J Funct Foods.

[CR3] Cho NH, Shaw JE, Karuranga S, Huang Y, Fernandes JDD, Ohlrogge AW, Malanda B (2018). IDF Diabetes Atlas: Global estimates of diabetes prevalence for 2017 and projections for 2045. Diabetes Res Clin Pr.

[CR4] Deedwania P (2018). Dangers of hypoglycemia in cardiac patients with diabetes: time to switch to safer. Newer Drugs.

[CR6] Khan MAR, Ara MH, Mamun SM (2018). Fatty acid composition and chemical parameters of Liza parsia. J Appl Chem.

[CR7] Kumar V, Prakash O, Kumar S, Narwal S (2011). α-glucosidase inhibitors from plants: A natural approach to treat diabetes. Pharmacogn Rev.

[CR8] Kutoh E, Kuto AN, Wada A, Kurihara R, Kojima R (2021). Regulations of free fatty acids and diabetic parameters in drug naïve subjects with type 2 diabetes treated with canagliflozin monotherapy. Drug Research.

[CR9] Mata R, Cristians S, Escandon-Rivera J-R, K., Rivero-Cruz, I. (2013). Mexican antidiabetic herbs: valuable sources of inhibitors of alpha -glucosidases. J Nat Prod.

[CR10] Miyazawa M, Yagi N, Taguchi K (2005). Inhibitory compounds of α-glucosidase activity from Arctium *lappa* L. J Oleo Sci.

[CR11] Nie K, Lu D, Sun B, Fang Y, Ning Z, Wang M (2020). Enzymatic hydration of linoleic acid followed with selective chain cleavage for biofuels and biomaterials production. J Biobased Mater Bio.

[CR12] Paul S, Hou CT, Sun CK (2010). α-Glucosidase inhibitory activities of 10-hydroxy-8(E)-octadecenoic acid: an intermediate of bioconversion of oleic acid to 7,10-dihydroxy-8(E)-octadecenoic acid. New Biotechnol.

[CR13] Pi F, Shinzawa H, Czarnecki MA, Iwahashi M, Suzuki M, Ozaki Y (2010). Self-assembling of oleic acid (cis-9-octadecenoic acid) and linoleic acid (cis-9, cis-12-octadecadienoic acid) in ethanol studied by time-dependent attenuated total reflectance (ATR) infrared (IR) and two-dimensional (2D) correlation spectroscopy. J Molr Struct.

[CR14] Rahim F, Ullah H, Javid MT, Wadood A, Taha M, Ashraf M, Shaukat A, Junaid M, Hussain S, Rehman W (2015). Synthesis, *in vitro* evaluation and molecular docking studies of thiazole derivatives as new inhibitors of α-glucosidase. Bioorg Chem.

[CR15] Su CH, Hsu CH, Ng LT (2013). Inhibitory potential of fatty acids on key enzymes related to type 2 diabetes. BioFactors.

[CR16] Teng H, Chen LJCRIFS (2016). α-Glucosidase and α-amylase inhibitors from seed oil: A review of liposoluble substance to treat diabetes. Crit Rev Food Sci.

[CR17] Tetko IV, Tanchuk VY (2002). Application of associative neural networks for prediction of lipophilicity in ALOGPS 2.1 program. J Chem Inf Comput Sci.

[CR18] Thao TTP, Bui TQ, Quy PT, Bao NC, Van Loc T, Van Chien T, Chi NL, Van Tuan N, Van Sung T, Nhung NTA (2021). Isolation, semi-synthesis, docking-based prediction, and bioassay-based activity of *Dolichandrone spathacea* iridoids: new catalpol derivatives as glucosidase inhibitors. RSC Adv.

[CR19] Trott O, Olson A (2009). Software News and Update AutoDock Vina: Improving the Speed and Accuracy of Docking with a New Scoring Function. J Comput Chem.

[CR20] Xu Y, Xie L, Xie J, Liu Y, Chen W (2019). Pelargonidin-3-O-rutinoside as a novel α-glucosidase inhibitor for improving postprandial hyperglycemia. Chem Commun.

[CR21] Yan Z, B., Engin, Eser, P., Kristensen, Z., and Engineering, G. (2020). Fatty acid hydratase for value-added biotransformation. Chinese J of Chem Phy.

[CR22] Yao L, Hammond E, Tong W (2008). Melting points and viscosities of fatty acid esters that are potential targets for engineered oilseed. J Am Oil Chem Soc.

[CR24] Zy A, Wei ZA, Ffa B, Yong ZA, Wk A (2014). α-Glucosidase inhibitors isolated from medicinal plants. Food Sci Hum Well.

