# Supplementary material for: Construction of fatty acid derivatives from rubber seed oil as α-glucosidase inhibitors based on rubber seed oil
Source: Bioresour Bioprocess. 2022 Mar 21;9(1):23. doi: 10.1186/s40643-022-00492-9 (PMC10992144; doi:10.1186/s40643-022-00492-9)
Supplement: Supplementary file 1 — Additional file 1. Supporting information. [file 40643_2022_492_MOESM1_ESM.docx]

Construction of fatty acid derivatives from rubber seed oil as α -glucosidase inhibitors based on rubber seed Oil

Jiahao Liu^1^, Renwei Zhang^1^, Kaili Nie^1^, Changsheng Liu^1, 2,^ *, Li Deng ^1,^ * and Fang Wang^1^

^1^ Beijing Bioprocess Key Laboratory and State Key Laboratory of Chemical Resource Engineering, College of Life Science and Technology, Beijing University of Chemical Technology (BUCT), Beijing, 100029, PR China

^2^ Sinovac Biotech Ltd, Beijing, China

*** Corresponding author Tel: +86-010-64414543; fax: +86-010-64416428.**

E-mail address: Changsheng Liu, liucs6972@sinovac.com; dengli@mail.buct.edu.cn (Li Deng).

[1. Methods 3](#_Toc91527079)

[1.1 Homologous modeling 3](#_Toc91527080)

[1.2 Expression of hydrase 3](#_Toc91527081)

[2. Figure 4](#_Toc91527082)

[Figure S1. Synthesis of hydroxy fatty acids 4](#_Toc91527083)

[Figure S2. Synthesis of oleate esters 4](#_Toc91527084)

[Figure S3. hydration of hydrolyzed fatty acids from rubber seed oil 5](#_Toc91527085)

[Figure S4. Fatty acid isopropyl esters 5](#_Toc91527086)

[Figure S5. FFAs and α-glucosidase complex binding site, prepared with LigPlot+ 6](#_Toc91527087)

[a) SA; b) HSA; c) OA; d) HOA 6](#_Toc91527088)

[Figure S6. Three-dimensional response surface 7](#_Toc91527089)

[3. Table 8](#_Toc91527090)

[Table S1. SPSS correlation analysis among inhibitory score, docking score and melting point of various FFA 8](#_Toc91527091)

[Table S2. The IC_50_, inhibitory score, affinity energy and melting point of various FFA 8](#_Toc91527092)

[Table S3. IC_50_, inhibitory score, affinity energy and melting point of hydroxy fatty acid 9](#_Toc91527093)

[Table S4. IC_50_, inhibitory score, affinity energy and melting point of oleate esters 9](#_Toc91527094)

[Table S5. composition of Hydrolyzed fatty acid by rubber seed oil 9](#_Toc91527095)

[Table S6. The IC_50_, carbon length, C=C bond number of various FFA 10](#_Toc91527096)

[Table S7. Results and analysis of Box-Behnken experiments for optimization of esterification reaction conditions 11](#_Toc91527097)

[Table S8: Variance analysis of response surface experiments results 12](#_Toc91527098)

# 1. Methods

## 1.1 Homologous modeling

The homologous modeling method of a-glucosidase was reported by Rahim et al., using the crystal structure of *Saccrhromyces cerevisiae* isomaltase (3AJ7.pdb) as template ([Rahim et al., 2015](#_ENREF_2)). The amino acid sequence of a-glucosidase selects the MAL12 a-glucosidase from Saccharomyces *cerevisiae* S288C (NC_001139.9, <http://www.uniprot.org/>). Homology modeling tools select Swiss-Model (<https://www.swissmodel.expasy.org/interactive>).

## 1.2 Expression of hydrase

The expression of Em-OAH enzyme referred to the reported method ([Demming et al., 2017](#_ENREF_1)). *Em-OAH* hydrase (PDB: 4UIR) was constructed at pET22b plasmid (Beijing Bio-Tool, Co., Ltd) with restriction enzyme cutting sites of HindIII and NdeI, shifting into *E. coli* BL21 (Beijing Bio-Tool, Co., Ltd) by thermal transition. *E. coli* was cultured in the Terrific broth (TB) medium (3L) in 5L fermentation tank at 37 ℃, 180 rpm. 0.1mM IPTG was added at OD600 of 0.5-0.7, incubating at 20 ℃, 180 rpm for another 20 h. Collecting the fermentation broth and centrifugating at 8000 rpm, 10 min, 4℃. The sediment was collected for overnight freeze-drying to obtain the lyophilized powder, which was used in the hydration reaction.

# 2. Figure


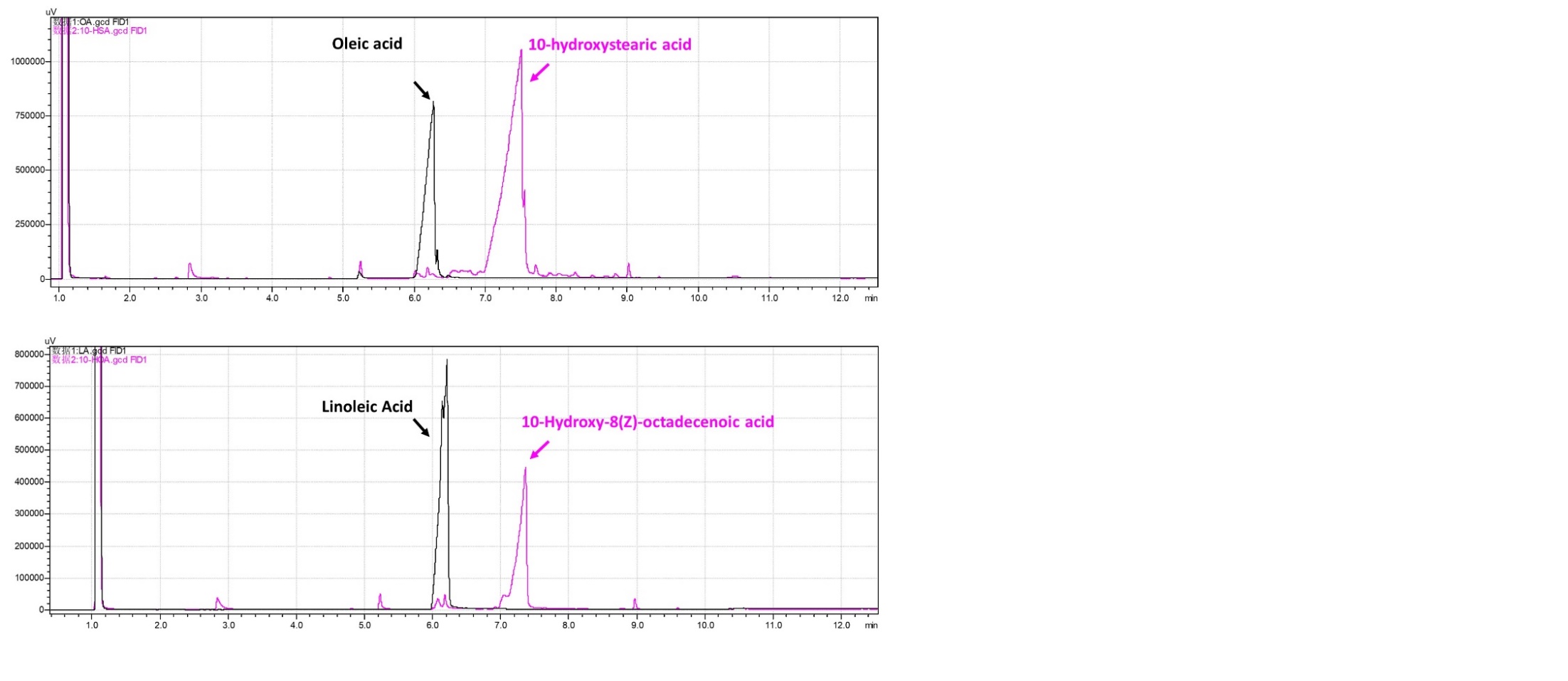


Figure S1. Synthesis of hydroxy fatty acids (10-HSA and 10-HOA).


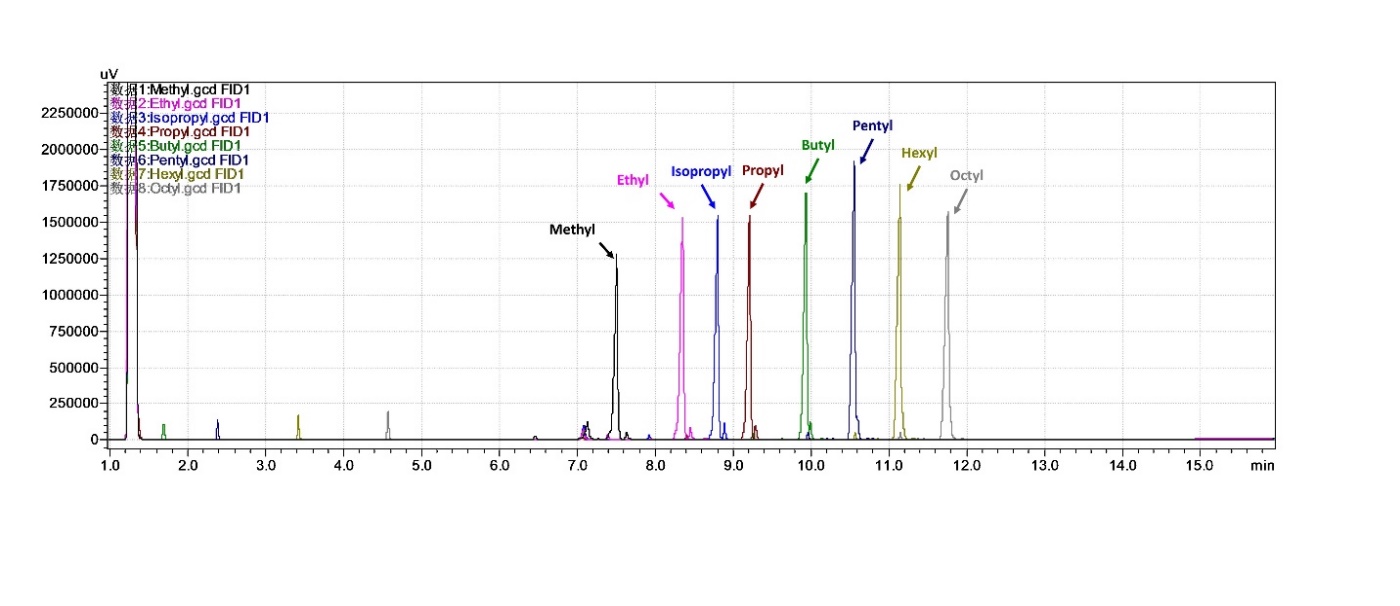


## Figure S2. Synthesis of oleate esters


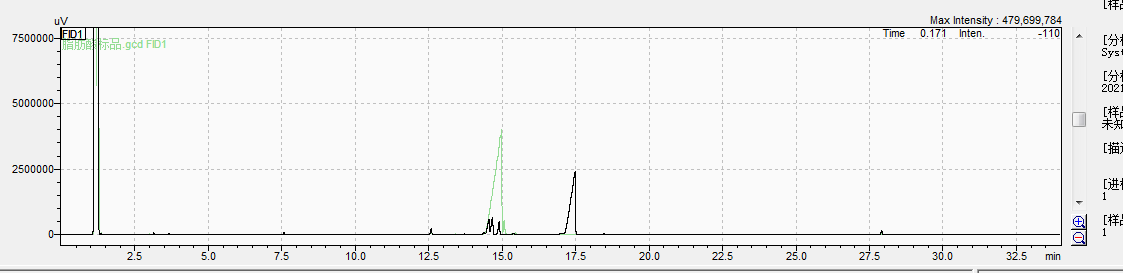


**FFAS**

**Hydroxy fatty acids**

Figure S3. hydration of hydrolyzed fatty acids from rubber seed oil (10-HSA+10-HOA+HLA)


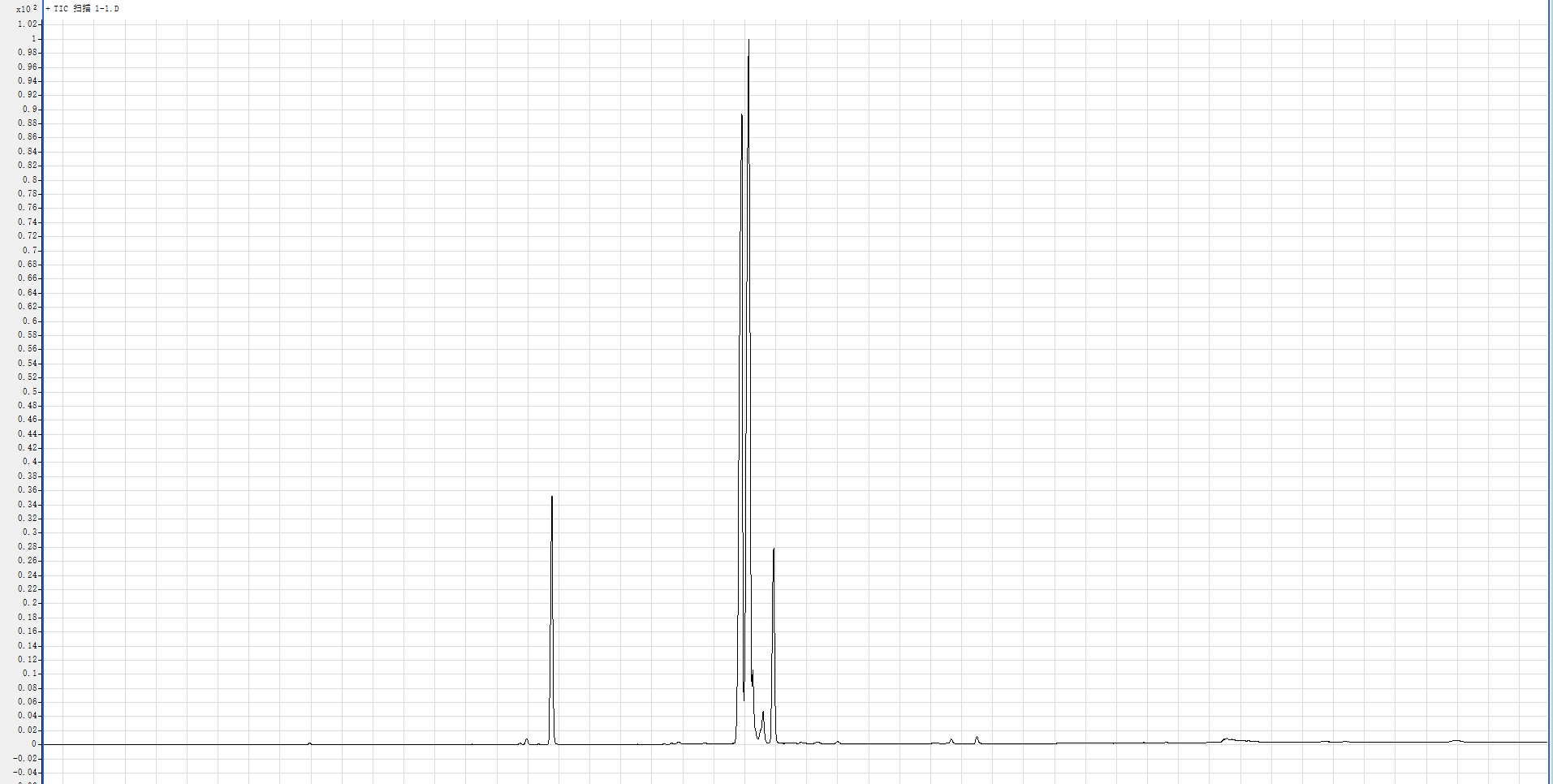


**1**

**2**

**3**

**4**

**5**


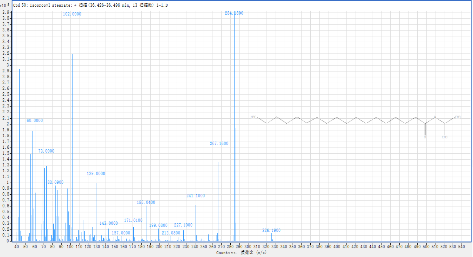

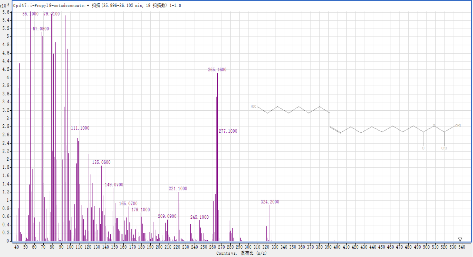


**1**


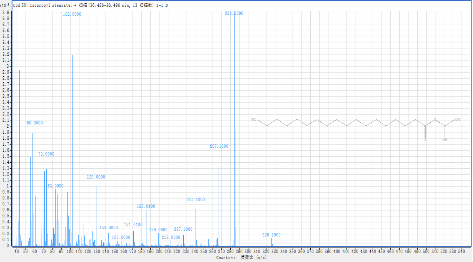


**2**

**3**


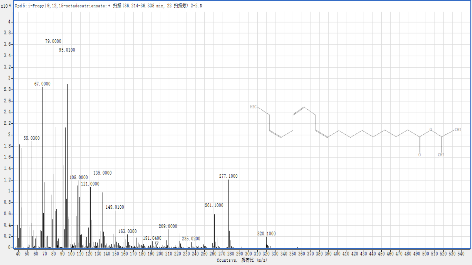


**4**


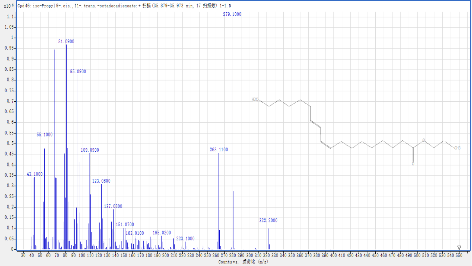


**5**

Figure S4. Fatty acid isopropyl esters 1) isopropyl palmitate. 2) isopropyl stearate 3) Isopropyl oleate. 4) Isopropyl linolenic acid. 5) isopropyl stearate


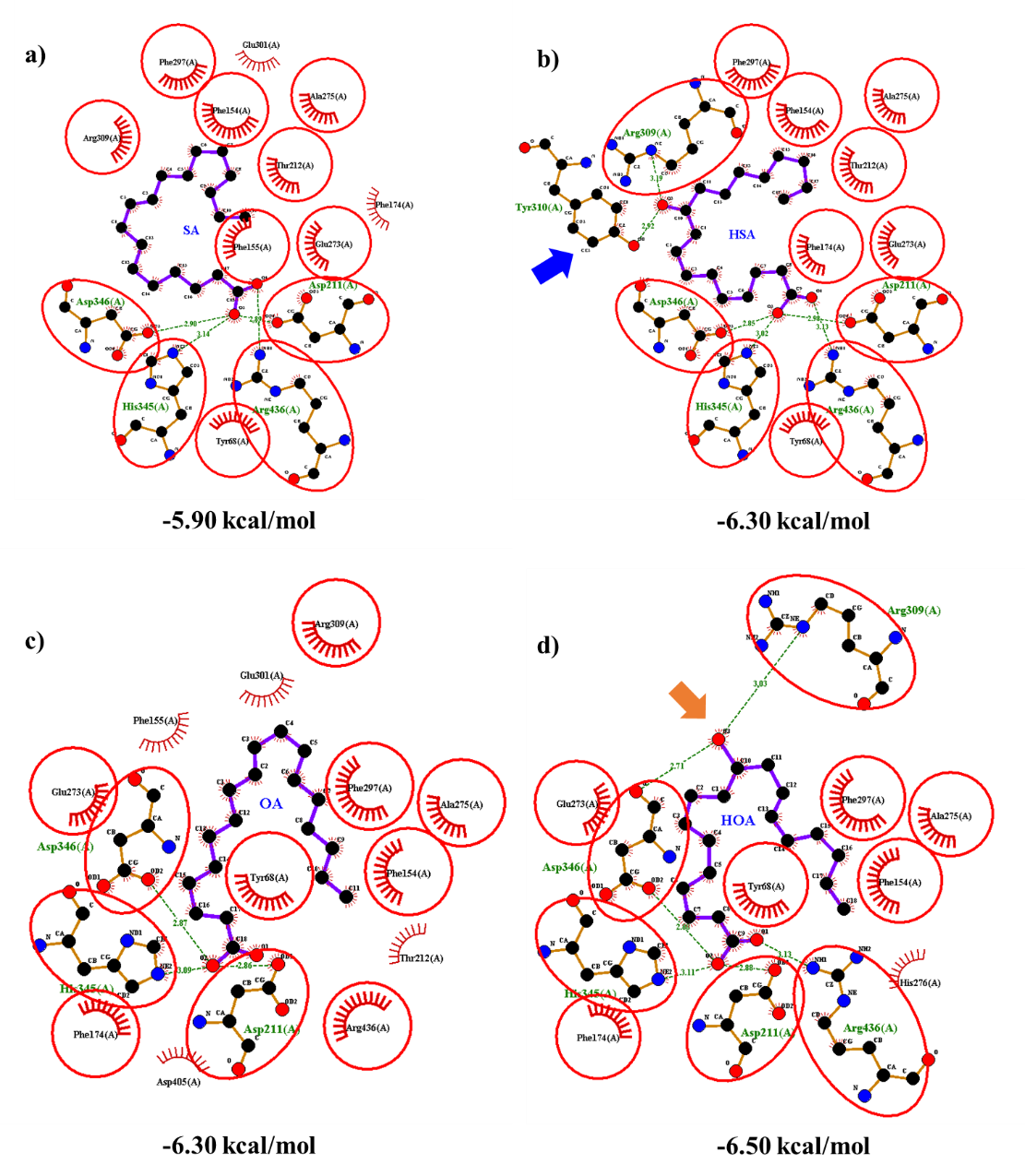


## Figure S5. FFAs and α-glucosidase complex binding site, prepared with LigPlot+

## a) SA; b) HSA; c) OA; d) HOA


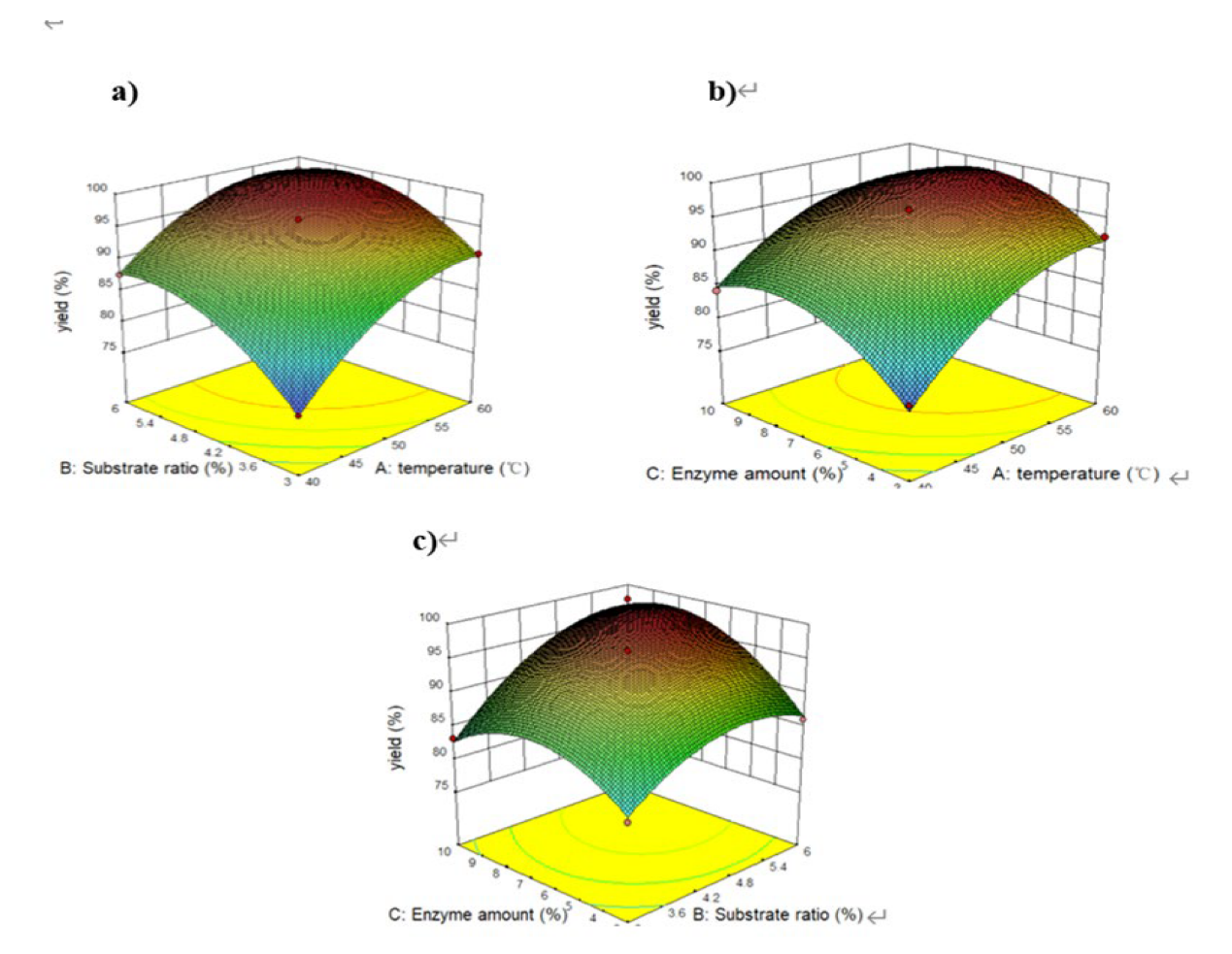


## Figure S6. Three-dimensional response surface

# 3. Table

## **Table S1.** SPSS correlation analysis among inhibitory score, docking score and melting point of various FFA

|  | **Docking score (kcal/mol)** | **Melting point (**°C) | **log P** |
| --- | --- | --- | --- |
| **Inhibitory score** | -0.87** | -0.88** | 0.35 |

**. P<0.01, significant correlation

## **Table S2.** The IC_50_, inhibitory score, affinity energy and melting point of various FFA

| Fatty acids | IC_50_ (μM) | Inhibitory score | Affinity energy  (kJ/mol) | Melting point (℃) | log P |
| --- | --- | --- | --- | --- | --- |
| C8:0 | 98.04±4.07 | 3.83 | -23.0 | 16.50 | 2.92 |
| C10:0 | 113.64±2.37 | 3.70 | -23.9 | 31.60 | 3.93 |
| C12:0 | 135.14±6.43 | 3.45 | -23.9 | 44.80 | 5.13 |
| C14:0 | 56.18±2.81 | 3.43 | -24.7 | 53.50 | 6.1 |
| C16:0 | 13.97±0.01 | 3.33 | -25.1 | 62.50 | 7.23 |
| C18:0 | 11.34±0.14 | 3.12 | -24.7 | 70.00 | 8.02 |
| *cis*-C18:1 | 0.81±0.04 | 4.51 | -26.4 | 13.00 | 7.68 |
| C18:2 | 0.60±0.01 | 4.99 | -27.2 | -5.00 | 7.11 |
| C18:3 | 0.54±0.01 | 5.34 | -28.5 | -11.00 | 6.65 |
| C20:5 | 0.48±0.01 | 6.70 | -32.2 | -47.40 | 6.83 |
| *trans*-C18:1 | ND | 3.92 | -26.4 | 44.00 | 7.68 |

## **Table S3.** IC_50_, inhibitory score, affinity energy and melting point of hydroxy fatty acid

| Fatty acids | IC_50_ (μM) | Inhibitory score | Melting point (℃) | Affinity energy  (kJ/mol) |
| --- | --- | --- | --- | --- |
| SA | 11.34±0.14 | 3.12 | 70.00 | -24.7 |
| 10-HSA | 1.71±0.02 | 3.21 | 81.00 | -26.4 |
| OA | 0.81±0.04 | 4.51 | 13.00 | -26.4 |
| 10-HOA | 0.39±0.01 | 4.79 | 5.50 | -27.2 |

## **Table S4.** IC_50_, inhibitory score, affinity energy and melting point of oleate esters

| Fatty acids | IC_50_ (μM) | Inhibitory score | Melting point (℃) | Affinity energy  (kJ/mol) |
| --- | --- | --- | --- | --- |
| OA | 0.81±0.04 | 1.23 | 4.51 | -26.4 |
| Methyl | 1.51±0.05 | 0.66 | 4.76 | -24.7 |
| Ethyl | 2.44±0.14 | 0.41 | 4.86 | -25.1 |
| Propyl | 1.75±0.04 | 0.57 | 4.89 | -24.3 |
| Butyl | 2.34±0.11 | 0.43 | 5.19 | -25.5 |
| Isopropyl | 0.60±0.01 | 1.67 | 5.22 | -25.5 |
| Pentyl | 3.21±0.17 | 0.31 | 5.00 | -25.1 |
| Hexyl | 4.16±0.21 | 0.24 | 5.01 | -25.1 |
| Octyl | 3.25±0.08 | 0.31 | 4.50 | -24.7 |

## **Table S5.** composition of Hydrolyzed fatty acid by rubber seed oil

| Fatty acids | C16:0 | C18:0 | C18:1 | C18:2 | C18:3 |
| --- | --- | --- | --- | --- | --- |
| Proportion (100%) | 9.545 | 6.69 | 28.368 | 37.635 | 16.672 |

## Table S6. The IC_50_, carbon length, C=C bond number of various FFA

| Fatty acids | IC50 (μM) | carbon length | C=C bond number |
| --- | --- | --- | --- |
| C8:0 | 98.04±4.07 | 8 | 0 |
| C10:0 | 113.64±2.37 | 10 | 0 |
| C12:0 | 135.14±6.43 | 12 | 0 |
| C14:0 | 56.18±2.81 | 14 | 0 |
| C16:0 | 13.97±0.01 | 16 | 0 |
| C18:0 | 11.34±0.14 | 18 | 0 |
| cis-C18:1 | 0.81±0.04 | 18 | 1 |
| C18:2 | 0.60±0.01 | 18 | 2 |
| C18:3 | 0.54±0.01 | 18 | 3 |
| C20:5 | 0.48±0.01 | 20 | 5 |
| trans-C18:1 | ND | 18 | 1 |

## Table S7. Results and analysis of Box-Behnken experiments for optimization of esterification reaction conditions

| Sequence Number | X1 | X2 | X3 | Y(Predicted) | Y(Actual) | Residual |
| --- | --- | --- | --- | --- | --- | --- |
| 1 | 40 | 4.5 | 3 | 77.038 | 78.294±2.51 | 1.256 |
| 2 | 60 | 4.5 | 3 | 92.196 | 92.885±5.32 | 0.689 |
| 3 | 50 | 3 | 3 | 80.611 | 80.836±4.89 | 0.225 |
| 4 | 50 | 4.5 | 6.5 | 96.081 | 96.335±3.69 | 0.254 |
| 5 | 50 | 4.5 | 6.5 | 95.422 | 96.076±2.56 | 0.654 |
| 6 | 60 | 3 | 6.5 | 90.856 | 91.114±1.20 | 0.258 |
| 7 | 40 | 3 | 6.5 | 75.618 | 76.644±8.53 | 1.026 |
| 8 | 40 | 6 | 6.5 | 87.554 | 89.116±2.69 | 1.562 |
| 9 | 50 | 6 | 3 | 86.071 | 86.319±7.56 | 0.248 |
| 10 | 50 | 4.5 | 6.5 | 95.081 | 95.627±3.63 | 0.546 |
| 11 | 50 | 4.5 | 6.5 | 94.081 | 94.559±5.41 | 0.478 |
| 12 | 50 | 4.5 | 6.5 | 95.077 | 95.533±6.35 | 0.456 |
| 13 | 50 | 3 | 10 | 83.211 | 83.808±0.25 | 0.597 |
| 14 | 60 | 6 | 6.5 | 97.943 | 98.208±5.44 | 0.265 |
| 15 | 50 | 6 | 10 | 97.738 | 98.286±4.45 | 0.548 |
| 16 | 40 | 4.5 | 10 | 84.213 | 84.428±4.26 | 0.215 |
| 17 | 60 | 4.5 | 10 | 94.191 | 94.445±5.41 | 0.254 |

## Table S8: Variance analysis of response surface experiments results

| source | Sum of Square | df | Mean Square | F Value | P Value  Prob>F | significance |
| --- | --- | --- | --- | --- | --- | --- |
| Model | 829.45 | 9 | 92.16 | 109.46 | <0.0001 | ** |
| X1 | 318.69 | 1 | 318.69 | 378.50 | <0.0001 | ** |
| X2 | 190.22 | 1 | 190.22 | 225.92 | <0.0001 | ** |
| X3 | 67.09 | 1 | 67.09 | 79.68 | <0.0001 | ** |
| X1X2 | 5.88 | 1 | 5.88 | 6.98 | 0.0333 |  |
| X1X3 | 6.03 | 1 | 6.03 | 7.16 | 0.0317 |  |
| X2X3 | 20.55 | 1 | 20.55 | 24.41 | 0.0017 | * |
| X1^2^ | 52.92 | 1 | 52.92 | 62.85 | <0.0001 | ** |
| X2^2^ | 55.01 | 1 | 55.01 | 65.33 | <0.0001 | ** |
| X3^2^ | 90.27 | 1 | 90.27 | 107.21 | <0.0001 | ** |
| Residual | 5.89 | 7 | 0.84 |  |  | ** |
| Lack of Fit | 3.79 | 3 | 1.26 | 2.40 | 0.2084 | Not significant |
| Pure Error | 2.10 | 4 | 0.53 |  |  |  |
| Cor Total | 835.35 | 16 |  |  |  |  |
|  |  |  |  | R^2^Adj=0.9929 |  |  |

**Figure legends**

Figure S1. Synthesis of hydroxy fatty acids **(10-HSA and 10-HOA).**

**Figure S2. Synthesis of oleate esters**

Figure S3. hydration of hydrolyzed fatty acids from rubber seed oil **(10-HSA+10HOA+HLA)**

Figure S4. Fatty acid isopropyl esters **1) isopropyl palmitate. 2) isopropyl stearate 3) Isopropyl oleate. 4) Isopropyl linolenic acid. 5) isopropyl stearate**

**Figure S5. FFAs and α-glucosidase complex binding site, prepared with LigPlot+**

1. **SA; b) HSA; c) OA; d) HOA**

**Figure S6. Three-dimensional response surface**

**Table legends**

Table S1. **SPSS correlation analysis among inhibitory score, docking score and melting point of various FFA**

Table S2. **The IC_50_, inhibitory score, affinity energy and melting point of various FFA**

Table S3. **IC_50_, inhibitory score, affinity energy and melting point of hydroxy fatty acid**

Table S4. **IC_50_, inhibitory score, affinity energy and melting point of oleate esters**

Table S5. **composition of Hydrolyzed fatty acid by rubber seed oil**

**Table S6. The IC_50_, carbon length, C=C bond number of various FFA**

**Table S7. Results and analysis of Box-Behnken experiments for optimization of esterification reaction conditions**

**Table S8: Variance analysis of response surface experiments results**
